# Metastatic serous borderline tumor with micro-invasive ovarian carcinoma presenting as a breast lump: A case report

**DOI:** 10.1097/MD.0000000000019383

**Published:** 2020-02-28

**Authors:** Fengge Dong, Xiao Xie, Xue Wei, Miao-miao Jiao, Junwu Duan, Linlin Pan, Lirong Bi, Zhimin Fan, Ming Yang

**Affiliations:** a Department of Breast Surgery; b Department of Pathology, First Hospital of Jilin University, Changchun, People's Republic of China.

**Keywords:** breast cancer, metastasis, ovarian tumors

## Abstract

**Rationale:**

Breast metastasis from serous borderline tumor with micro-invasive carcinoma of ovary is a very rare condition. The breast lump as the only clinical presentation is rarely seen in ovarian carcinoma, which may lead to be misdiagnosed, and the mechanism of breast metastasis from ovarian tumors in early stage still needs to be explored. Differentiation from primary breast cancer and extramammary malignancy is crucial because the treatment and prognosis are significantly different.

**Patient concerns:**

A 33-year-old female presented with a painless, movable, 1.0 × 1.0 cm lump in the upper outer quadrant of the right breast for a month.

**Diagnoses:**

Breast metastasis of serous borderline tumor with micro-invasive ovarian carcinoma confirmed by pathology and immunohistochemistry.

**Interventions:**

The patient underwent lumpectomy, bilateral ovarian tumor stripping operation and prophylactic chemotherapy.

**Outcomes:**

No signs of recurrence have been detected in 1.5 years of follow-up.

**Lessons:**

Distant metastasis may occur in early stage of ovarian carcinoma. It is important to determine the origin of the primary tumor and develop an effective treatment strategy for patients. Imaging findings and pathological diagnostic criteria are important to accurately differentiate between metastasis and primary breast lesions, which may improve the patient's outcomes.

## 1 Introduction

Ovarian carcinoma is the most commonly disseminated intraperitoneally, but breast metastasis cannot be seen frequently in early stage. Metastasis of ovarian tumors to breast is a very rare condition.^[1,2]^ Ascites, abdominal pain, abdominal distension are the most common symptom of ovary carcinoma,^[3]^ however, the breast lump as the first clinical presentation for ovarian carcinoma is rarely seen. Breast metastasis from serous borderline tumor with micro-invasive carcinoma of ovary has never been reported in literature. Differentiation from primary breast cancer and extramammary malignancy is crucial because the treatment is significantly different.^[4]^ In this report, a case of serous borderline tumor with micro-invasive carcinoma of ovary that metastasizes to breast is presented. Interestingly, the breast lump was the only clinical presentation and prior to this, the patient had no any systemic symptoms.

## 2 Case report

This study was approved by the Ethics Committee and the Institutional Review Board of the First Hospital of Jilin University.

**Figure 1:**
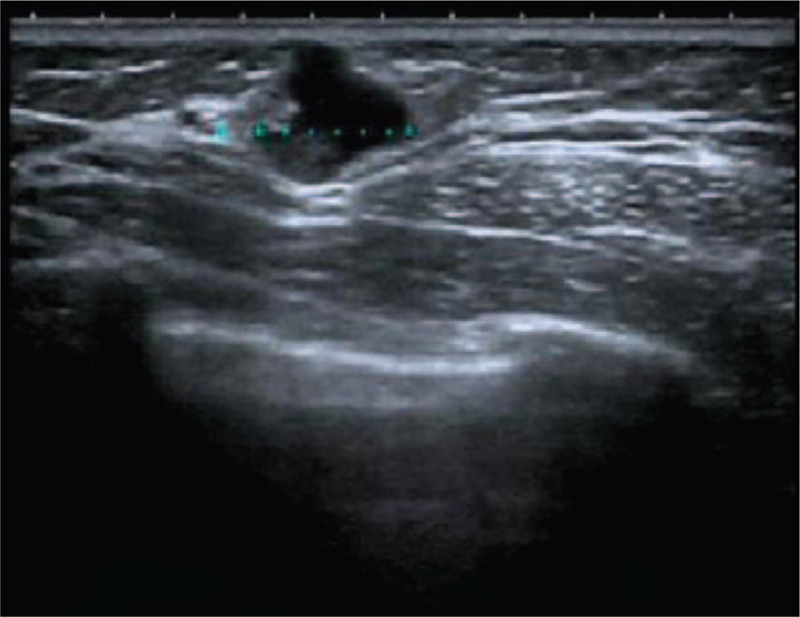
Breast ultrasound revealed a mixed echogenic mass containing cystic and solid components and measured 10.8 mm × 10.1 mm.

**Figure 2:**
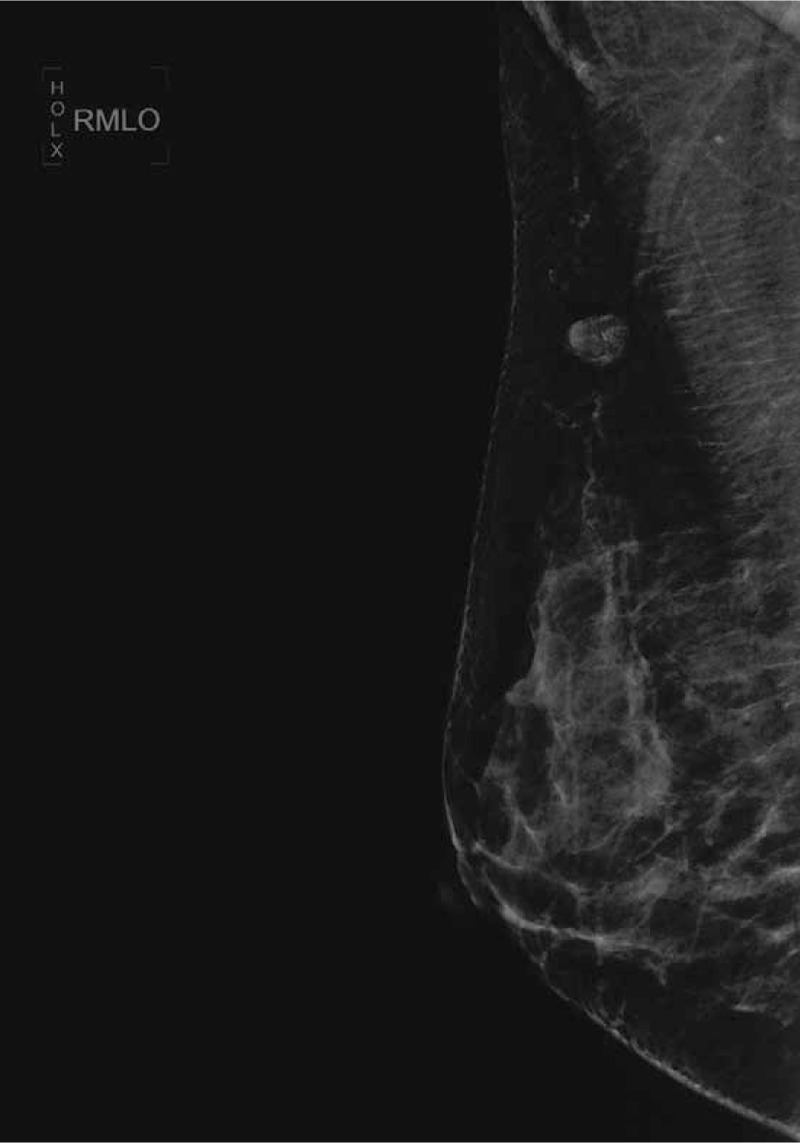
Mammography showed a slightly high-density lump of the right breast.

**Figure 3:**
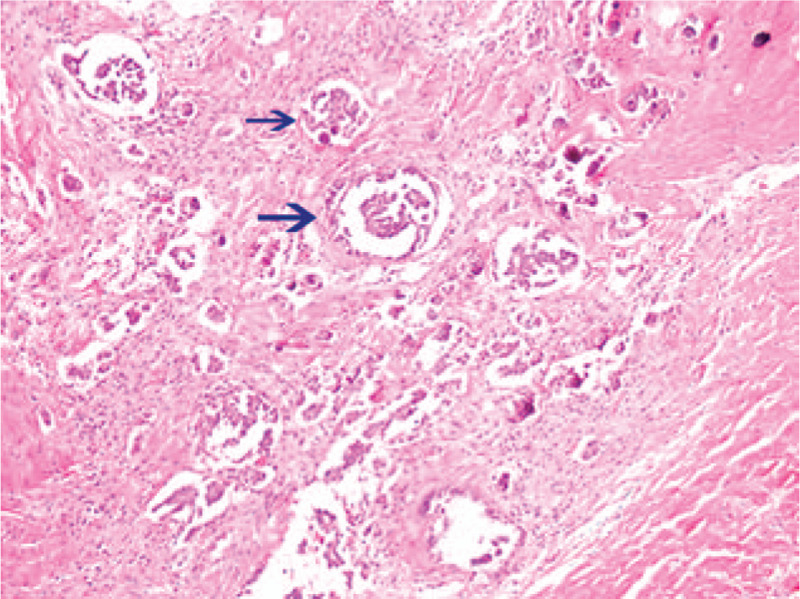
Clinicopathological examination of the resected breast tissue which revealed invasive papillary carcinoma with psammoma bodies (blue arrow) (hematoxylin and eosin staining; ×200).

**Figure 4:**
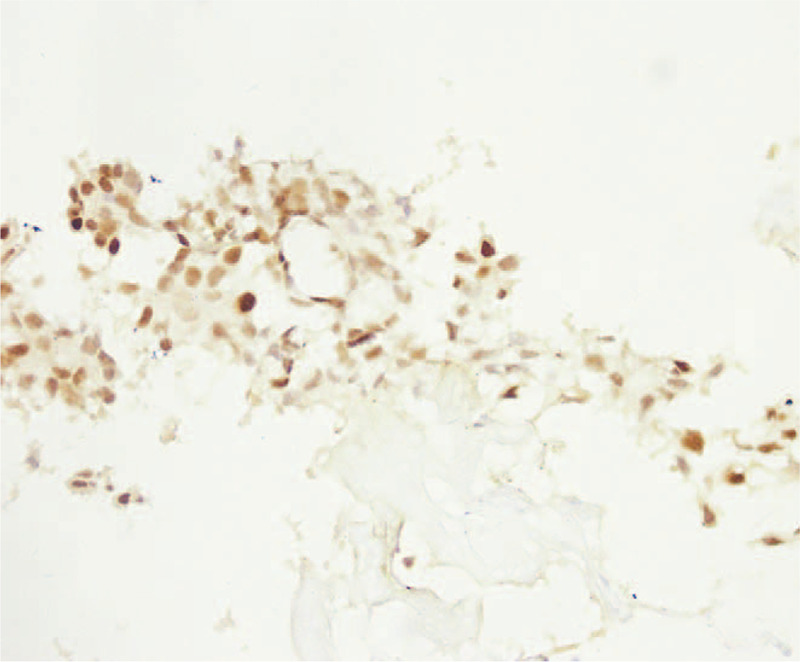
Immunohistochemical staining showed diffuse expression of PAX8 in breast tissue (magnification ×100).

**Figure 5:**
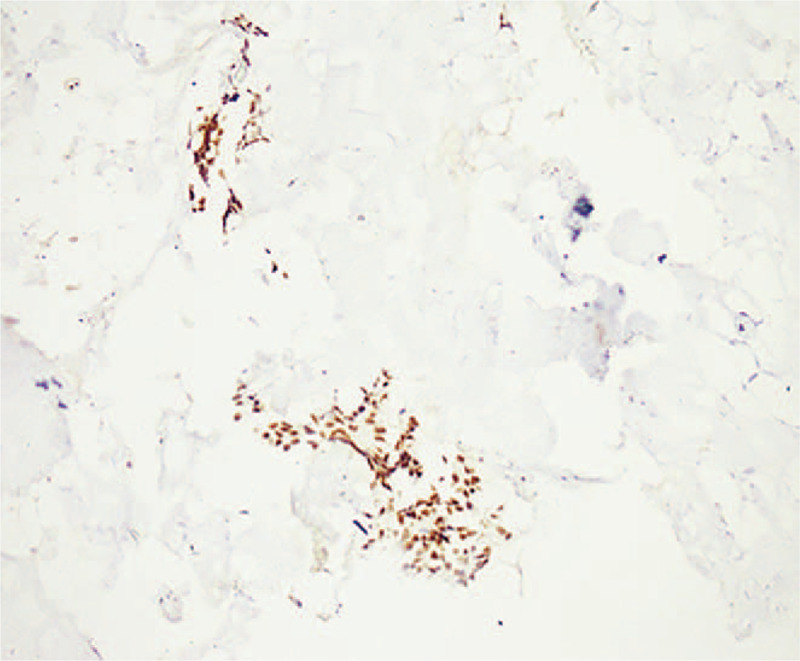
Immunohistochemical staining showed diffuse expression of WT-1 in breast tissue (magnification ×100).

**Figure 6:**
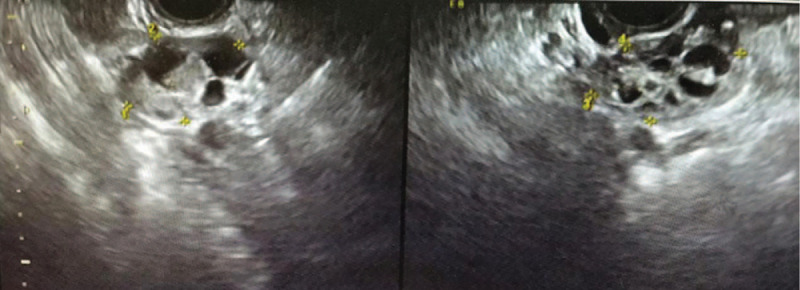
Vaginal ultrasound showing that the maximum diameters of the lumps in the right and left ovaries were 46 mm × 34 mm and 46mm × 29 mm respectively, which were also cystic and solid components.

In December 2017, a 33-year-old female, previously healthy female with a family history of breast cancer (aunt, sister of her father), who presented with a painless, movable lump in the upper outer quadrant of the right breast of one month duration. Ultrasound revealed a mixed echo lump containing cystic and solid components; the lump was 10.8 mm × 10.1 mm in size (Fig. 1). No suspicious axillary lymph nodes were involved. Mammography showed a slightly high-density lump in the upper outer quadrant of the right breast (Fig. 2). The patient underwent lumpectomy. Pathological result revealed papillary carcinoma with psammoma bodies (Fig. 3). Immunohistochemistry showed the following: estrogen receptor (ER, +60%), progesterone receptor (PR, +70%), epidermal growth factor receptor-2 (HER-2, 0), proliferation marker Ki67 (+60%), PAX8 (+) (Fig. 4) and WT-1 (+)(Fig. 5). Histological and immunohistochemistry analysis confirmed that the malignancy originated from extramammary tumor, which might originate from the ovary or lung. A chest computed tomography (CT) scan showed normal and vaginal ultrasound revealed multiple lumps in bilateral ovaries. The maximum diameters of the lumps in the right and left ovaries were 46 mm × 34 mm and 46mm × 29 mm respectively, which were also cystic and solid components (Fig. 6). Then, positron emission tomography/computed tomography (PET-CT) showed that there were no other metastatic lesions. Serum tumor markers such as CA125 were also normal.

**Figure 7:**
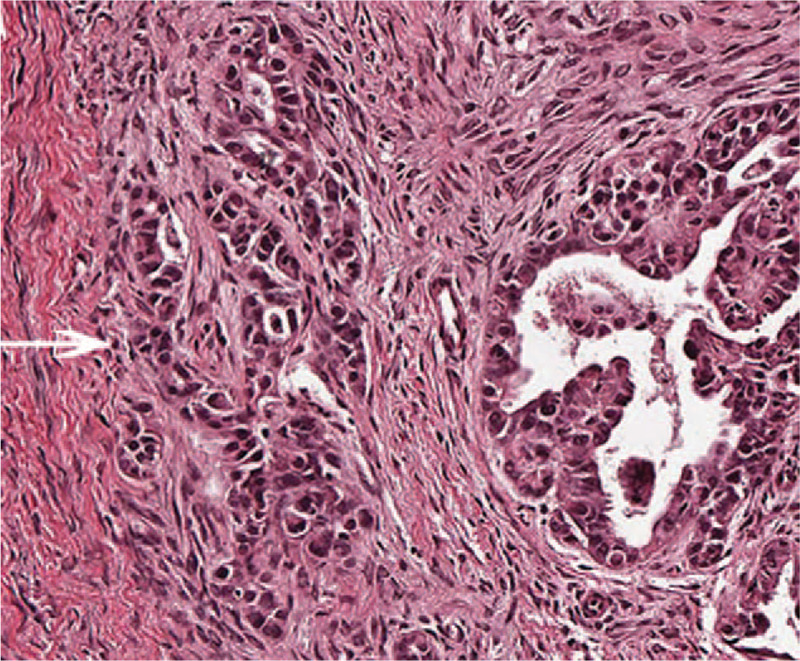
Clinicopathological examination of the resected ovarian tissue on the left-side showing serous borderline tumor with micro-invasive carcinoma. The maximum diameter was approximately about 1 mm (white arrow) (hematoxylin and eosin staining; ×200).

In January 2018, the patient underwent laparotomy and bilateral ovarian tumor stripping operation. The laparotomy showed a multitude of papilliform excrescences in the bilateral ovary. Following specimen collection and pathology revealed the diagnosis of serous borderline tumor with micro-invasive carcinoma of ovary (Fig. 7). Immunohistochemistry showed the following results: ER (+); PR (+); HER-2 (0); Ki-67 (approximately 30%+); Cytokeratin7 (+); PAX-8(+); P53 (<30%+); WT-1 (+). Breast tumors were evaluated with the slides of primary ovarian carcinoma counterparts, which confirmed the ovary origin of the lesion. Then the patient received prophylactic chemotherapy every 3 weeks and completed 6 cycles (240 mg of Paclitaxel and 500 mg of Carboplatin). No signs of recurrence have been detected in 1.5 years of follow-up.

## 3 Discussion

In 2018, there are 2.1 million new cases of breast cancer among females. Breast cancer is not only the most commonly diagnosed cancer but also the leading cause of cancer deaths among women.^[5]^ However, metastasis from extramammary malignancy to the breast is a rare condition, accounting for only 0.3% of all breast malignancies.^[3]^ Furthermore, the patient in this case study is diagnosed with borderline tumor with micro-invasive carcinoma of ovary with low malignant potential, which appears to be even more rare. Breast metastatic lesions are typically described as appearing quite benign on both mammographic and clinical examinations. In addition, the lumps are usually superficial, rarely adhere to the surrounding tissues, and has no skin adhesion.^[6]^

The mechanism of breast metastasis from ovarian cancer is still unclear and may be related to dissemination, hematogenous or lymphatic. Sippo et al^[4]^ considered that hematological disseminated metastases often developed as a circumscribed mass, whereas lymphatic dissemination often presented as diffuse breast edema and skin thickening. Tumors that blocked lymph vessel might lead to lymphedema and enlargement. Hence, lymphatic metastasis could be more similar to inflammatory breast cancer.

There are 35 published literatures of breast metastasis from ovarian carcinoma between 1981 and 2019, ranging from 14 to 81 years old (Table 1). Thirty cases of ovarian tumors metastasizing to breast were serous carcinoma,^[2,7–29]^ 2 cases were carcinoid tumor,^[30,31]^ 2 cases were clear-cell carcinoma,^[32,33]^ 2 cases were borderline tumor,^[20,34]^ 2 cases were small cell carcinoma,^[35,36]^ 1 was mucinous tumor of low malignant potential,^[37]^ 1 was dysgerminoma,^[38]^ 1 was granulosa cell tumor^[39]^ and 1 was endometrioid carcinoma.^[40]^ Thirty-one cases (73.2%) of these patients with breast metastasis had the history of ovarian carcinoma. Wadhwa et al^[26]^ reported an asymptomatic patient who developed a breast lump 1 month after ovarian surgery. Panse et al^[7]^ described a ovarian cancer patient who presented with a metastatic breast mass 8 year later. However, the outcome of the patient was not available. Seven cases had primary ovarian cancer and breast lumps concurrently.^[9,12,13,15,27,36,40]^ This article reported a case of ovary borderline tumor with micro-invasive carcinoma presenting as the breast lump.

**Table 1:**
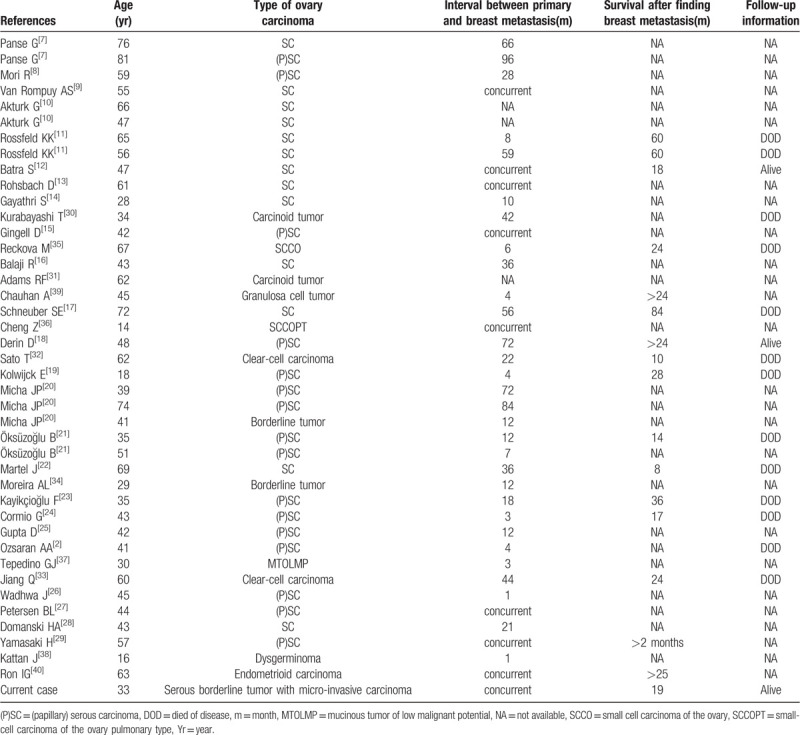
Reported cases of breast metastasis from ovary carcinoma.

Imaging studies and pathological diagnostic criteria are important to accurately differentiate between metastatic and primary breast cancer because the treatment and prognosis are significantly different. Mammography is valuable for differential diagnosis of primary and secondary breast cancers. The typical X-ray picture of secondary breast malignancy shows a clear, dense mass without calcification, distortion or thickening of the skin.^[28]^ There is a good correlation between imaging findings and physical examination in evaluating the size of lump. Primary breast tumors usually have significant pro-connective tissue hyperplasia reaction. Therefore, the size of the lump on imaging is smaller than those on physical examination while the metastatic lesions are usually consistent with clinical imaging.^[41]^ Immunohistochemical markers are also significant and the combined use of multiple markers can provide more sufficient diagnostic evidence.^[3]^ WT-1, PAX-8, and GCDFP-15 play a major role in differentiation between primary breast tumors and breast metastasis from ovarian tumors. WT-1 is mainly expressed in ovarian cancer, and less than 10% of primary breast cancers are positive for WT-1, which are usually weakly expressed or focally expressed.^[7,9,42]^ PAX8 is a pivotal transcription factor in ovary and regulates the expression of WT-1, which is admitted with higher sensitivity and specificity than WT-1. The positive rate of PAX-8 in serous ovarian carcinoma is as high as 79%, which is one of the reliable indicators to differentiate primary ovary cancer from breast cancer. GCDFP-15 is a highly sensitive and specific marker of breast carcinoma marker, which may help to identify the primary site of origin. In a recent immunohistochemical study, GATA 3 is more sensitive than GCDFP-15 in detecting the origin of primary breast cancer. GCDFP-15 and GATA 3 can be used to distinguish between primary and metastatic breast malignancy.^[7,43]^ The positive expression of WT-1, PAX8 and ER in breast specimens made us suspect that the primary carcinoma originated from the ovary. Then the pathology of ovarian neoplasms confirmed the ovary origin of the lesion. Comprehensive medical history and radiological findings are not sufficient to differentiate between primary or metastatic involvement of the breast. Pathology and immunohistochemistry are gold standard for diagnosis of metastasis.

There are no standard treatment guidelines for breast metastasis from ovarian cancer in early stage. Simple excisional biopsy and systemic therapy for the carcinoma constitute the preferred manner of treatment for today.^[6,20,21,29]^ We reviewed the 35 published literatures that breast metastasized from ovarian tumors. Most patients received surgery and only a minority received adjuvant chemotherapy. Of these, 13 cases died of disease, 2 cases were alive with no recurrence at the end of last follow up and 26 cases lacked of follow-up information. Breast metastasis from ovarian carcinoma in early stage may reflect poor prognosis in patients.^[44]^ Survival has been observed to be between 2 and 84 months after the occurrence of breast metastasis.^[17,29]^

To our knowledge, this is the first case of serous border tumor with micro-invasive carcinoma of ovary simultaneously metastasizes to the breast reported to date. We emphasize the accurate diagnosis of metastatic breast cancer and differentiation from primary breast cancer which are meaningful for patients’ management, avoiding misdiagnosis and developing appropriate systemic treatment protocols.^[45]^ However, the mechanism of serous borderline tumor with micro-invasive carcinoma of ovary metastasizing to breast in early stage still needs to be explored.

## 4 Conclusions

In conclusion, it is important to determine the origin of primary tumor and develop an effective treatment strategy for breast metastasis from ovarian tumors. Imaging findings and pathological diagnostic criteria are important to accurately differentiate between metastatic and primary breast lesions because of the treatment and prognosis are significantly different.

## Author contributions

**Conceptualization:** Xiao Xie.

**Data curation:** Fengge Dong, Junwu Duan.

**Investigation:** Xue Wei, Miao-miao Jiao, Linlin Pan.

**Supervision:** Ming Yang.

**Visualization:** Lirong Bi, Zhimin Fan.

**Writing – original draft:** Fengge Dong.

**Writing – review & editing:** Ming Yang.
